# Supplementary material for: Environmentally adjusted δ13C thresholds for accurate detection of C4 plant consumption in Europe
Source: Commun Earth Environ. 2025 Dec 18;6(1):1021. doi: 10.1038/s43247-025-03031-4 (PMC12714575; doi:10.1038/s43247-025-03031-4)
Supplement: Supplementary file 2 — Supplementary Information [file 43247_2025_3031_MOESM2_ESM.pdf]

Supplementary information to

Environmentally adjusted $\delta^{13}C$ thresholds for accurate detection of C_4 plant consumption in Europe

Margaux L. C. Depaermentier[1,*,$], Michael Kempf[2,*,$], Giedrė Motuzaitė Matuzevičiūtė[1]

[1] Faculty of History, Vilnius University, Vilnius, Lithuania.

[2] Department of Environmental Sciences, University of Basel, Basel, Switzerland.

*Corresponding authors: margaux.depaermentier@if.vu.lt; michael.kempf@unibas.ch

[$]These authors contributed equally to this paper.

ORCID MLCD: 0000-0002-1801-3358; margaux.depaermentier@if.vu.lt
ORCID MK: 0000-0002-9474-4670; michael.kempf@unibas.ch
ORCID GMM: 0000-0001-9069-1551; giedre.motuzaite@gmail.com

Supplementary figures S1 to S10 (pages 2 to 11).

Captions of supplementary data 1 and 2 (pages 10 to 12).

Supplementary table S1 (page 12).

Supplementary reference (page 12).

Supplementary figures S1 to S10

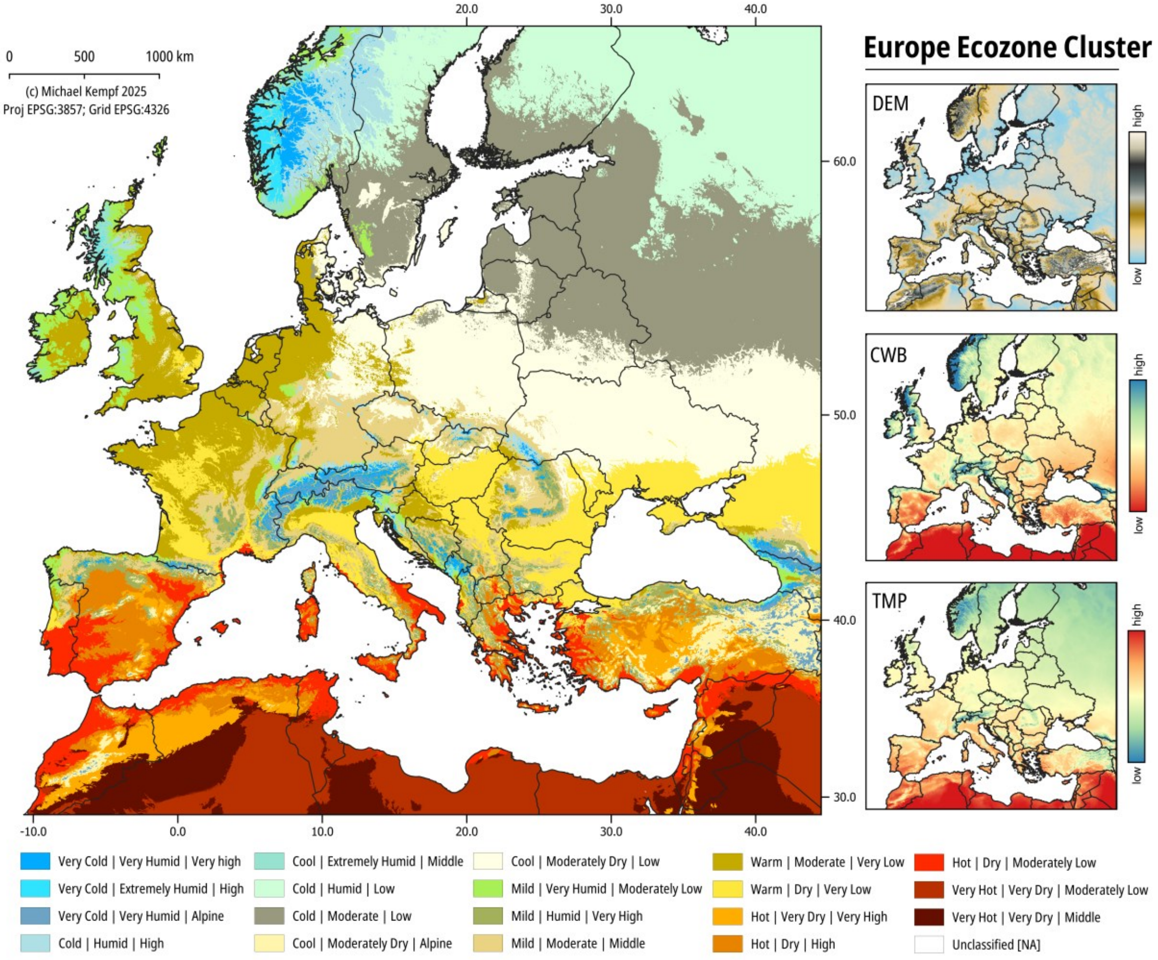

Fig. S1 | Modelled ecozone clusters over Europe. 20 clusters based on temperature (TMP), moisture availability (CWB), and topography (DEM) were defined using *k-means* cluster analysis (with *k*=20), including unclassified NA values (i.e., inland water). See the methods and material section for a description of the open source TMP, CWB and DEM data and their provenience. The ecozones are described and numbered in Tab. 1. Figure by Michael Kempf, created using the open source R and QGIS software.

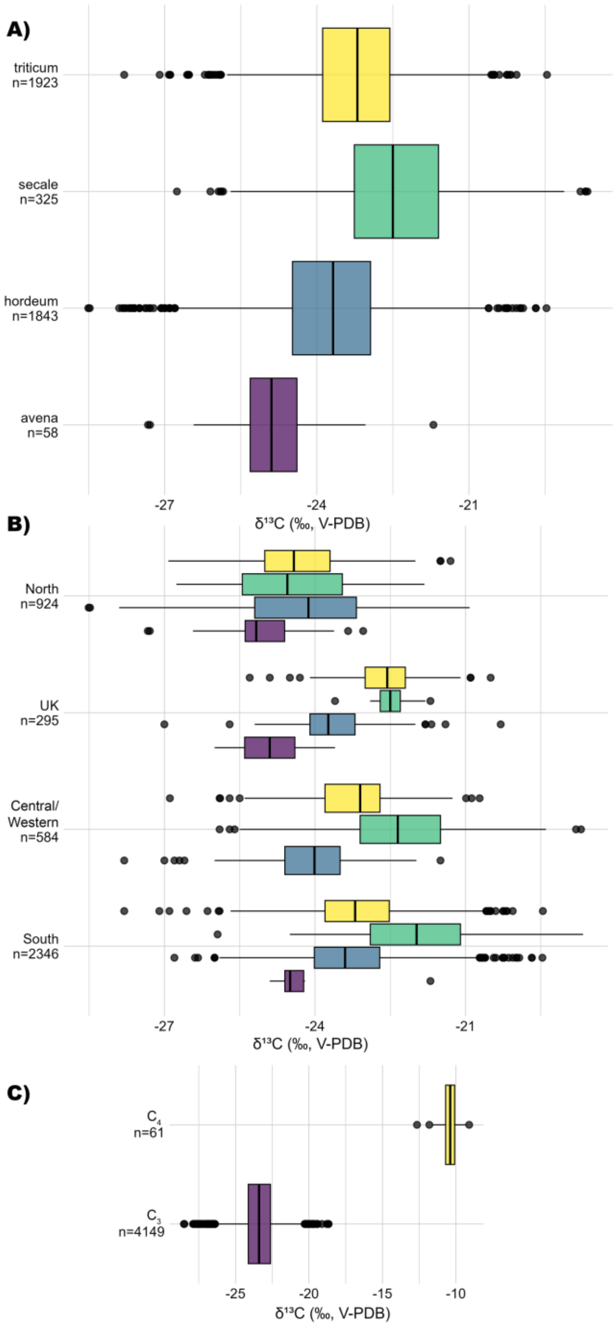

Fig. S2 | Species-specific δ¹³C values in Europe. (A) Main species *δ*¹³C within the entire sample. (B) Main species *δ*¹³C within the various parts of Europe (see (a) for the cereal's color code). (C) C_3 and C_4 plants *δ*¹³C values from Europe. Boxplots are defined in Fig. 2. The results of the related statistical tests are listed in Supplementary data 2. Figure by Margaux L. C. Depaermentier, created using the open source R software.

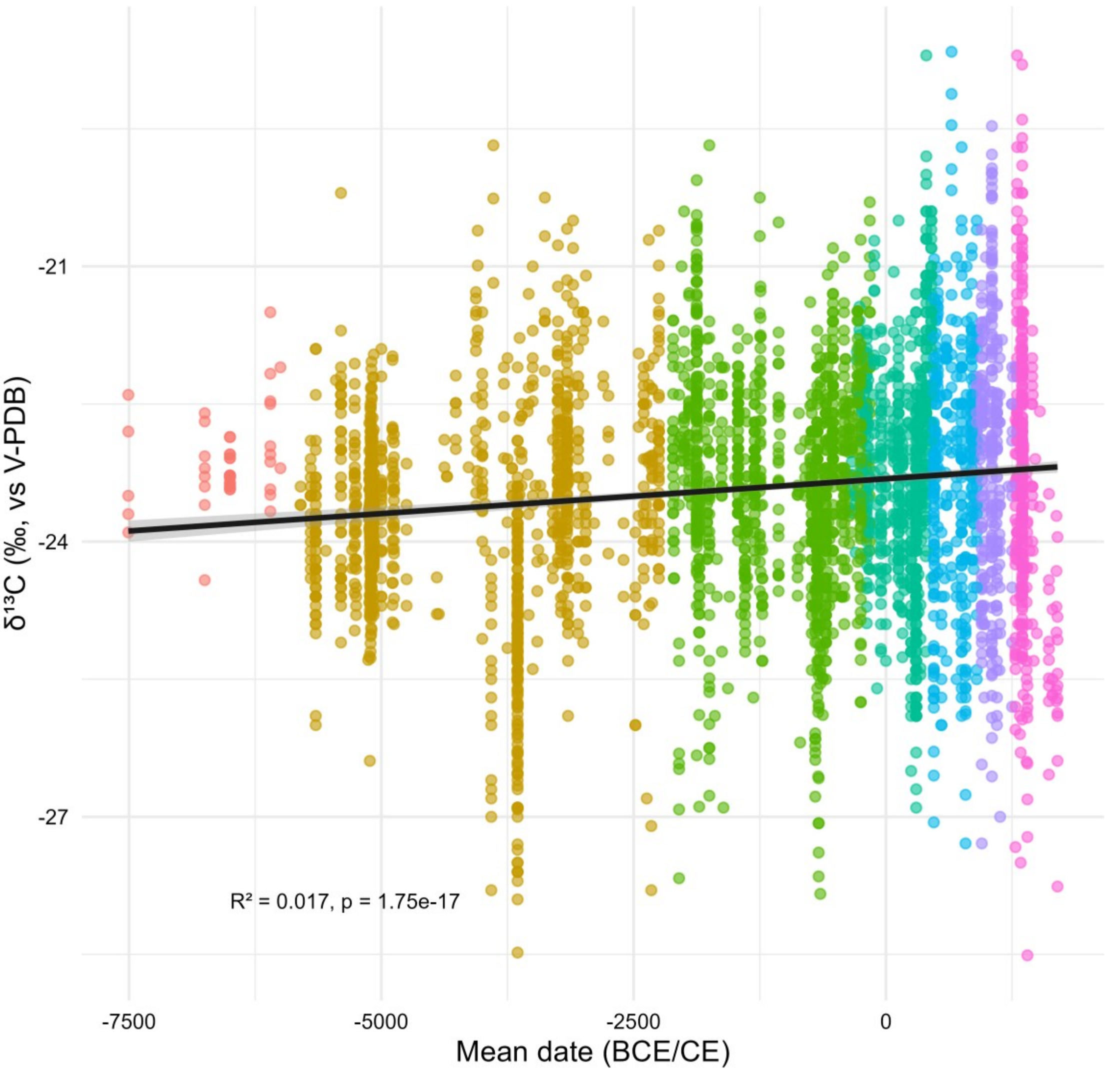

Fig. S3 | Temporal evolution of charred C_3 grains $\delta^{13}C$ values in Europe. The mean date is derived from radiocarbon dating when available or from the archaeological datation range. The line and its shade represent the smoothed linear model calculated for the entire sample. The color-code reflects the main climatic phases: red: Early Holocene (8000-6000 BCE), gold: Holocene Climatic Optimum (6000-2000 BCE), green: Late Holocene Cooling (2000-500 BCE), turquoise: Roman Warm Period (250 BCE - 400 CE), blue: Late Antique Little Ice Age (500-900 CE), purple: Medieval Warm Period (950-1200 CE), and pink: Little Ice Age (1300-1850 CE). The results of the related statistical tests are listed in Supplementary data 2. Figure by Margaux L. C. Depaermentier, created using the open source R software.

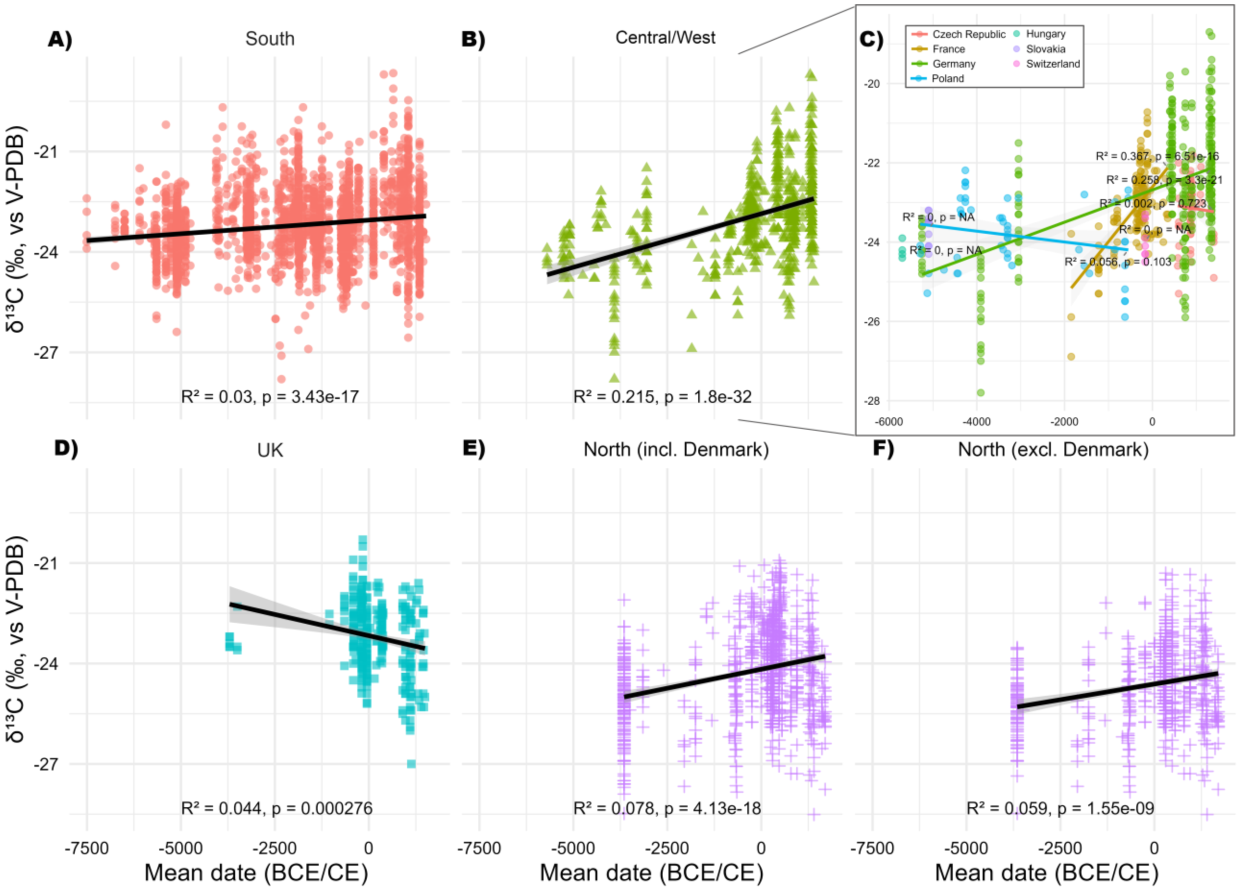

Fig. S4 | Temporal evolution of charred C_3 grain's $\delta^{13}C$ values in Europe. (A) Southern Europe, (B) Central/Western Europe, (C) country-specific patterns within Central/Western Europe, (D) the UK, (E) Northern Europe including Denmark, (F) Northern Europe excluding Denmark. The lines and their grey shade represent the smoothed linear model calculated for each region or country. The results of the related statistical tests are listed in Supplementary data 2. Figure by Margaux L. C. Depaermentier, created using the open source R software.

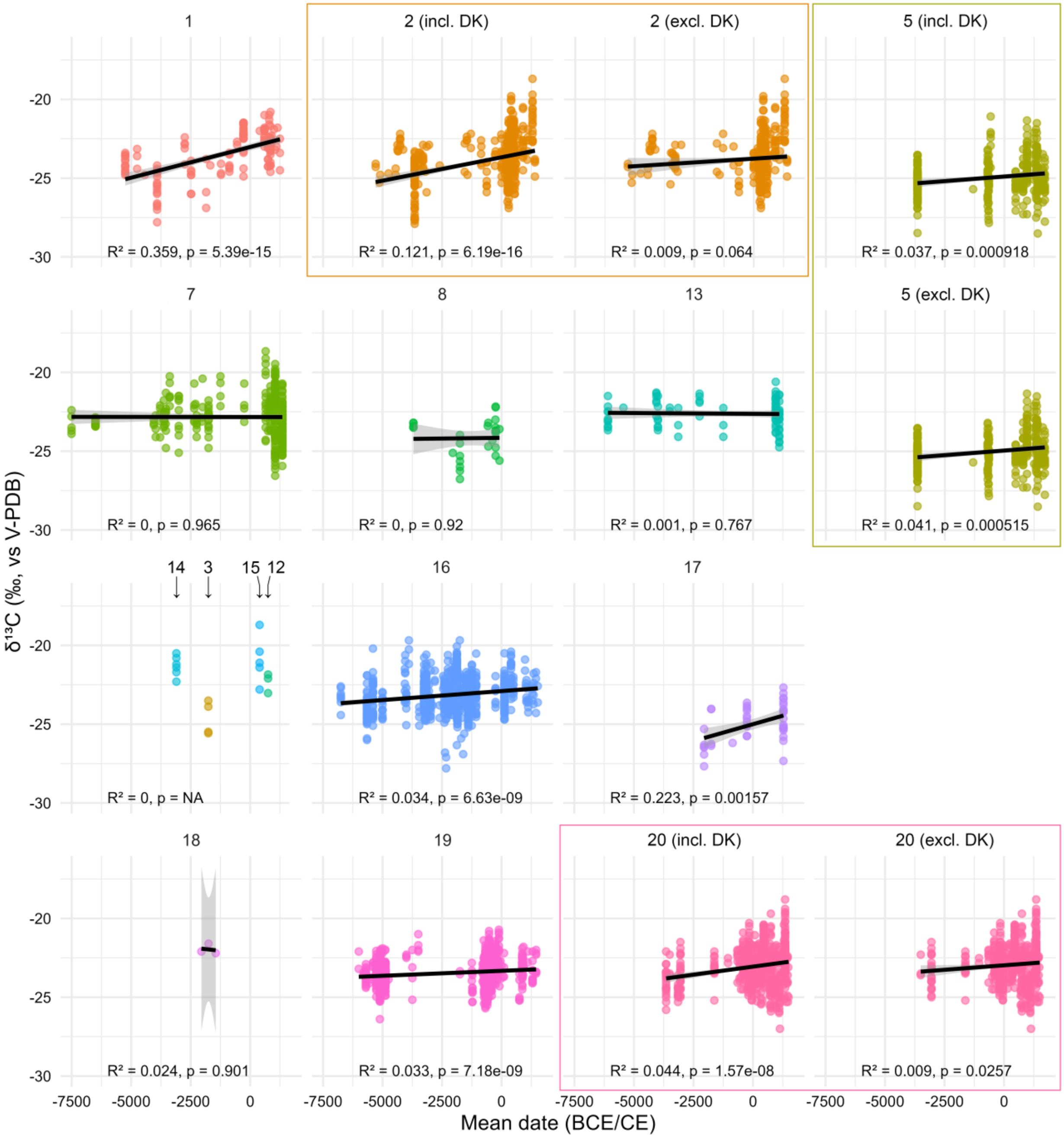

Fig. S5 | Temporal evolution of charred C_3 grains $\delta^{13}C$ values within the modelled European ecozones. The ecozones are described in Tab. 1. The lines and their shade represent the smoothed linear models calculated for each ecozones. The samples from Denmark are scattered over three ecozones (2, 5, and 20) and the linear model was calculated for each ecozone once including and once excluding the Danish samples due to the important temporal shift within this sample (see Fig. S7). The results of the related statistical tests are listed in Supplementary data 2. Figure by Margaux L. C. Depaermentier, created using the open source R software.

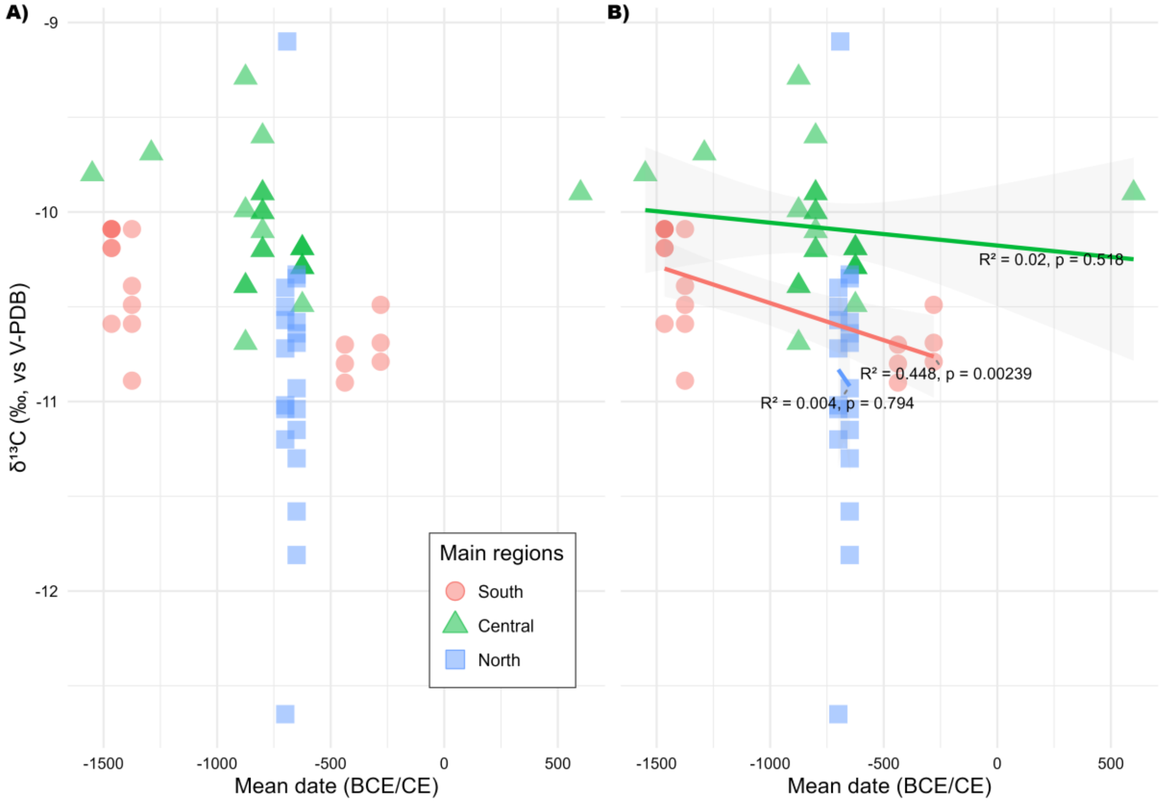

Fig. S6 | Temporal evolution of charred C_4 grains $\delta^{13}C$ values in Europe. The mean dates are derived from the published archaeologically determined chronological ranges. (A) There is hardly any chronological overlap between the C_4 grains from the various regions. (B) The linear models investigating the relation between $\delta^{13}C$ and the mean date are thus not considered significant (see Supplementary data 2 for the results of the statistical tests). Figure by Margaux L. C. Depaermentier, created using the open source R software.

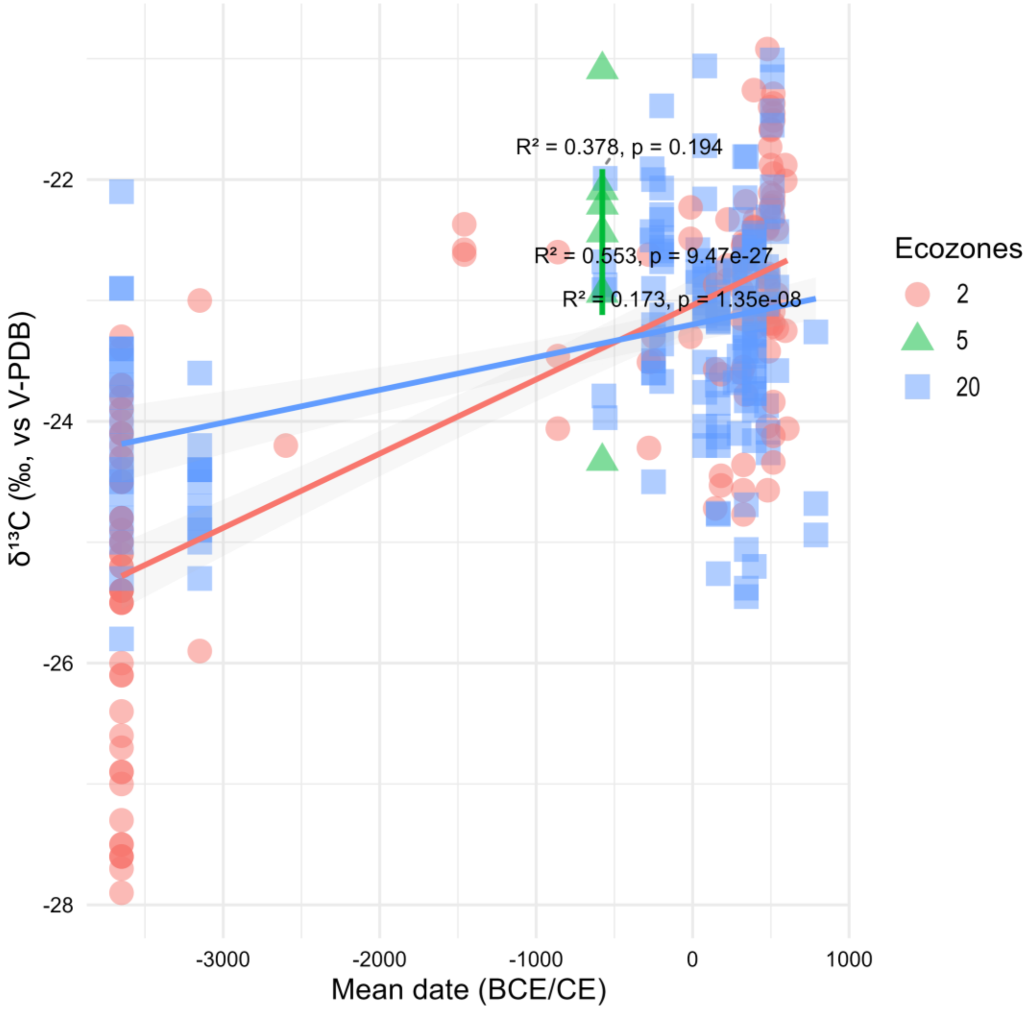

Fig. S7 | Charred C_3 grains $\delta^{13}C$ values in Denmark. The colors and shape represent the location of each grain within one of the three ecozones from Denmark. The ecozones are defined in Tab. 1. The mean date is derived from radiocarbon dating if available or from the archaeologically estimated range if no ^{14}C date is available. The lines and their grey shades represent the linear models calculated for each ecozone over time. The results of the related statistical tests are listed in Supplementary data 2. Figure by Margaux L. C. Depaermentier, created using the open source R software.

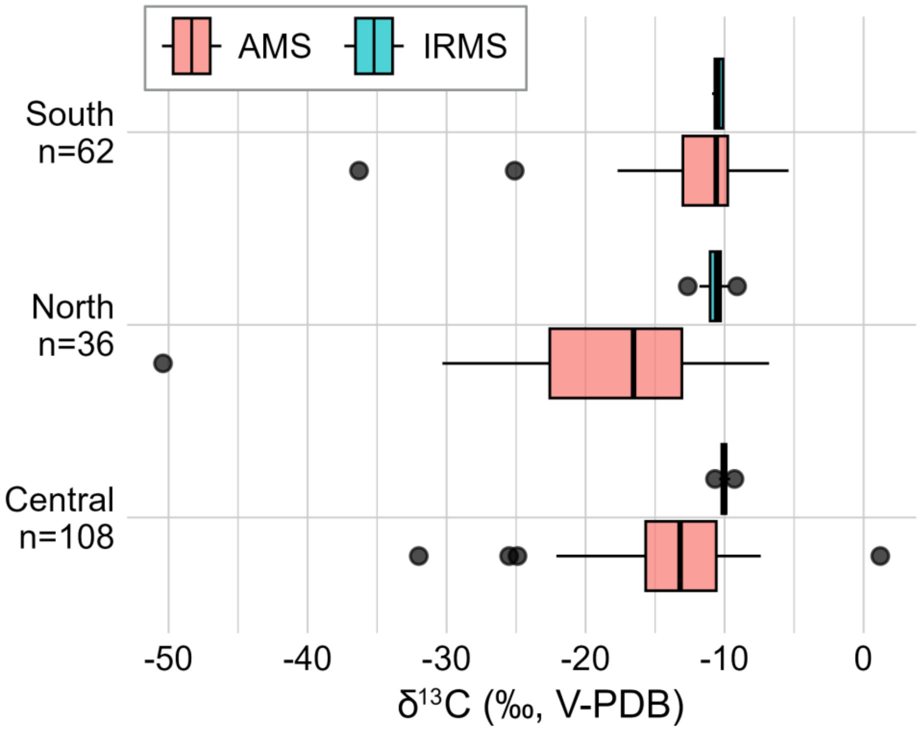

Fig. S8 | Charred C_4 grains $\delta^{13}C$ values from IRMS and AMS. The AMS values are derived from radiocarbon measurements[1] and show a much larger range than the IRMS data. Figure by Margaux L. C. Depaermentier, created using the open source R software.

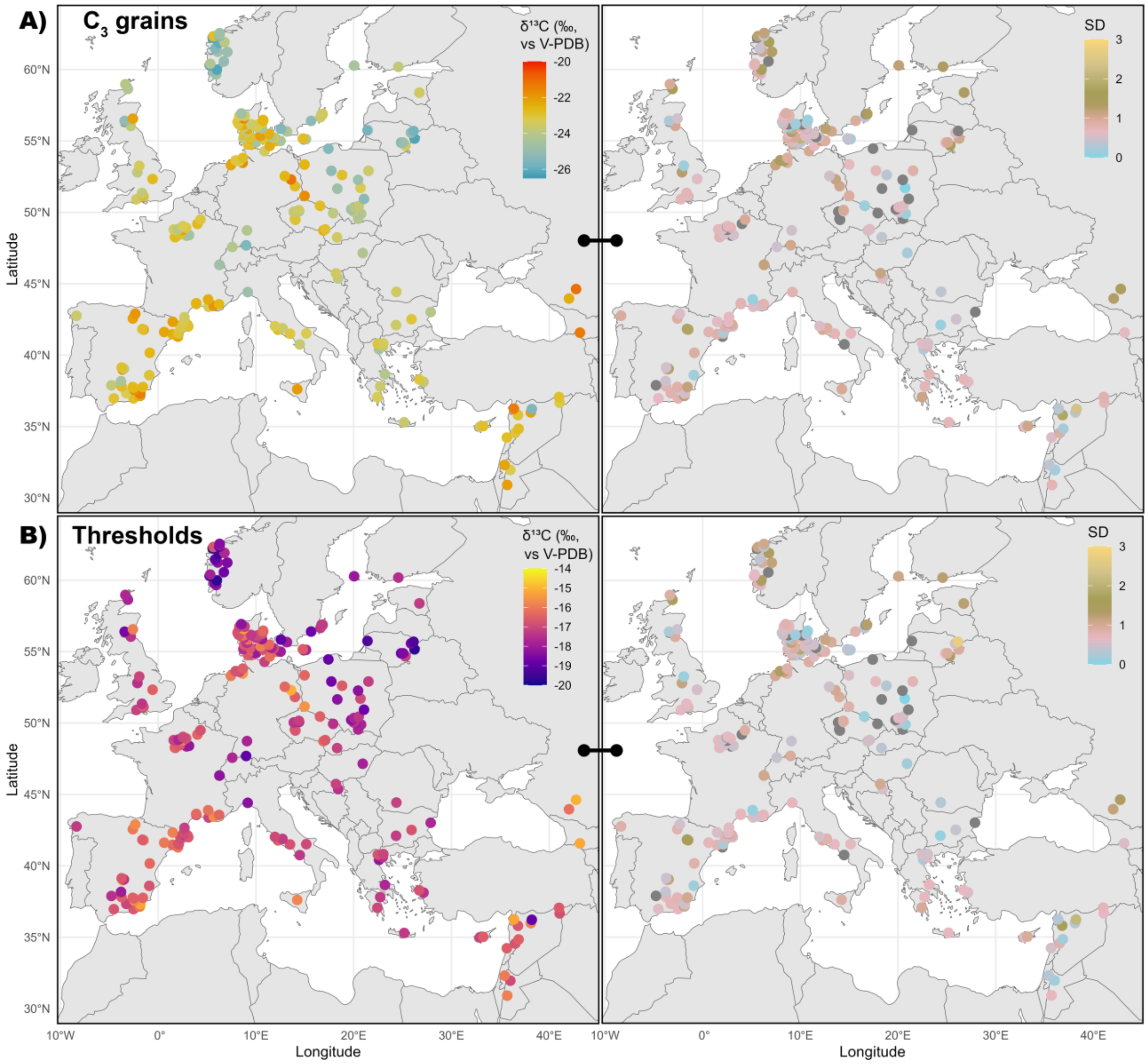

Fig. S9 | Point-based approach for baseline $\delta^{13}C$ values and estimated threshold values for C_4 diet identification in mammal collagen at all sites. (A) Median C_3 grain $\delta^{13}C$ values (left) and related SD (right). (B) Median estimated threshold $\delta^{13}C$ values for mammal collagen (left) and related SD (right) based on a theoretical 100%-grain-based-diet. The mean, median, SD and MAD values for each site are listed in Supplementary data 1. The same maps including inly sites with n≥10 grains are in Fig. 5. Figure by Margaux L. C. Depaermentier, created using the open source R software.

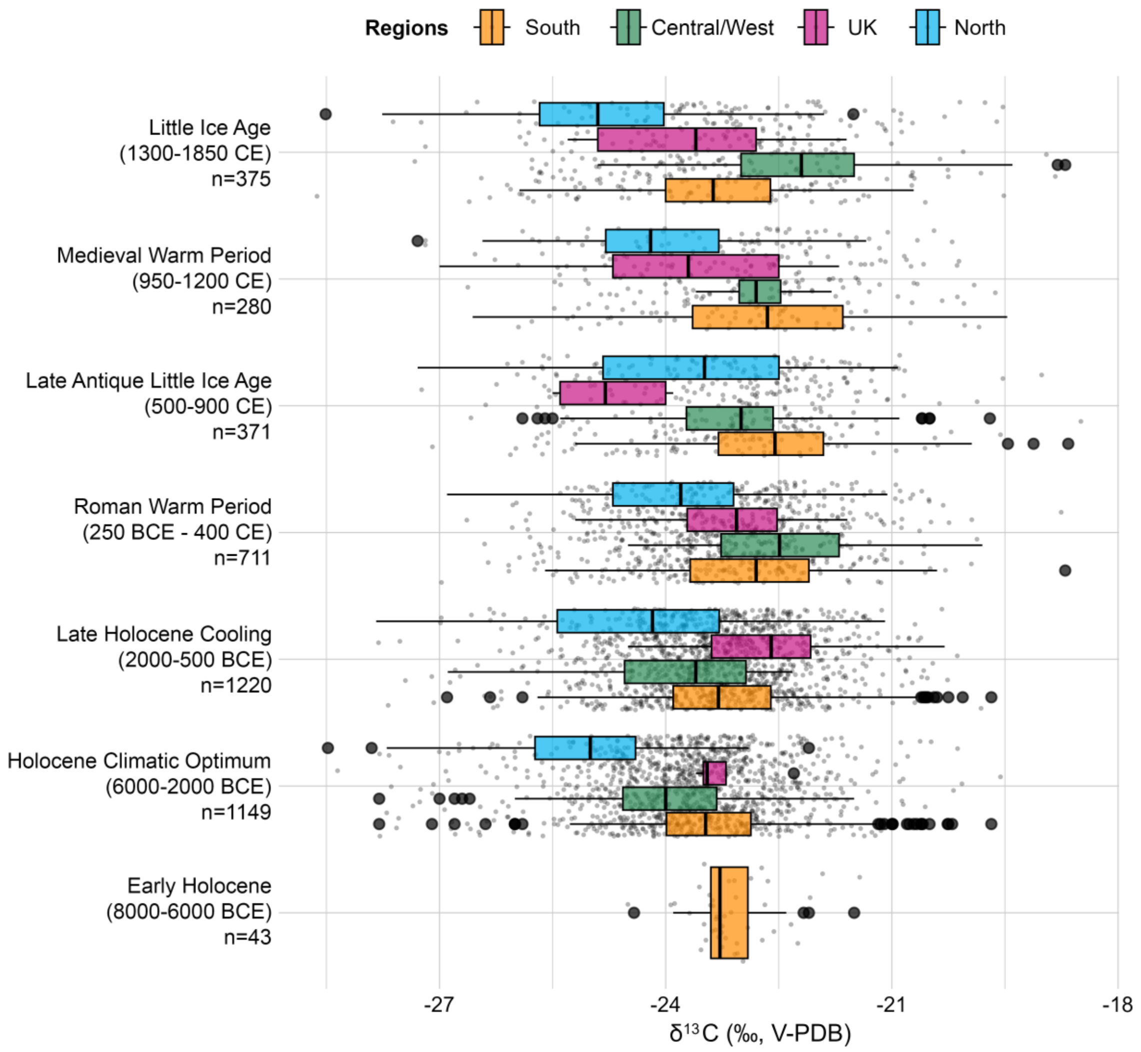

Fig. S10 | C_3 grain $\delta^{13}C$ values in each region over the various climatic phases. The results of the one-way ANOVA tests for each region are available from Supplementary data 2. Figure by Margaux L. C. Depaermentier, created using the open source R software.

Captions of supplementary data 1 and 2

[Uploaded as Excel file]

Supplementary data 1 | Summary statistics for the C_3 and C_4 grains $\delta^{13}C$ values and the theoretical collagen $\delta^{13}C$ values based on various diets. The data is uploaded as separate Excel file due to its large size.

[Uploaded as Excel file]

Supplementary data 2 | Results of the statistical tests ran in this study. The data is uploaded as separate Excel file due to its large size.

Supplementary table S1

Ecozone cluster	n samples	Mean theoretical threshold $\delta^{13}C$ (‰)	Median theoretical threshold $\delta^{13}C$ (‰)	MAD for theoretical threshold $\delta^{13}C$ (‰)	1 SD for theoretical threshold $\delta^{13}C$ (‰)	comment
**1**	140	-17.15	-17.03	1.29	1.26	
**2**	511	-17.48	-17.51	1.22	1.28	
**3**	4	-18.26	-18.32	1.13	0.96	small sample size
**5**	294	-18.55	-18.59	0.97	1.12	
**7**	338	-16.56	-16.63	1.10	1.22	
**8**	29	-17.83	-17.48	0.90	1.07	
**12**	3	-16.09	-15.89	0.33	0.56	small sample size
**13**	86	-16.38	-16.31	0.81	0.78	
**14**	6	-15.19	-15.17	0.60	0.58	small sample size
**15**	5	-14.82	-15.03	0.95	1.37	small sample size
**16**	964	-16.84	-16.88	0.85	0.90	
**17**	42	-18.57	-18.67	1.33	1.08	
**18**	3	-15.77	-15.89	0.13	0.29	small sample size
**19**	1007	-17.14	-17.15	0.70	0.74	
**20**	717	-16.74	-16.71	0.99	1.05	

Tab. S1 | Summary statistics for the C_3 and C_4 grains $\delta^{13}C$ values and the theoretical collagen $\delta^{13}C$ values for C_4 input identification.

Supplementary references

1. Filipović, D. *et al.* New AMS 14C dates track the arrival and spread of broomcorn millet cultivation and agricultural change in prehistoric Europe. *Sci Rep* 10, 13698; 10.1038/s41598-020-70495-z (2020).
