# Supplementary material for: Environmentally adjusted δ13C thresholds for accurate detection of C4 plant consumption in Europe
Source: Commun Earth Environ. 2025 Dec 18;6(1):1021. doi: 10.1038/s43247-025-03031-4 (PMC12714575; doi:10.1038/s43247-025-03031-4)
Supplement: Supplementary file 3 — Description of Additional Supplementary Files [file 43247_2025_3031_MOESM3_ESM.pdf]

Description of Additional Supplementary Files

File name: Supplementary Data 1
Description: Summary statistics for the C3 and C4 grains δ13C values and the theoretical collagen δ13C values based on various diets.

File name: Supplementary Data 2
Description: Results of the statistical tests ran in this study.
